# Naturally occurring Vpr inhibitors from medicinal plants of Myanmar

**DOI:** 10.1007/s11418-017-1104-7

**Published:** 2017-07-05

**Authors:** Nwet Nwet Win, Hla Ngwe, Ikuro Abe, Hiroyuki Morita

**Affiliations:** 1 Institute of Natural Medicine, University of Toyama, 2630-Sugitani, Toyama, 930-0194 Japan; 2 Department of Chemistry, University of Yangon, Yangon, 11041 Myanmar; 3 Graduate School of Pharmaceutical Sciences, The University of Tokyo, 7-3-1 Hongo, Bunkyo-ku, Tokyo, 113-0033 Japan

**Keywords:** Viral protein R, TREx-HeLa-Vpr cells, *K. pulchra*, *P. javanica*, Isopimarane diterpenoids, Picrasane quassinoids

## Abstract

Human immunodeficiency virus type-1 (HIV-1) is a lentiviral family member that encodes the retroviral Gag, Pol, and Env proteins, along with six additional accessory proteins, Tat, Rev, Vpu, Vif, Nef, and Vpr. The currently approved anti-HIV drugs target the Pol and Env encoded proteins. However, these drugs are only effective in reducing viral replication. Furthermore, the drugs’ toxicities and the emergence of drug-resistant strains have become serious worldwide problems. Resistance eventually arises to all of the approved anti-HIV drugs, including the newly approved drugs that target HIV integrase (IN). Drug resistance likely emerges because of spontaneous mutations that occur during viral replication. Therefore, new drugs that effectively block other viral components must be developed to reduce the rate of resistance and suppress viral replication with little or no long-term toxicity. The accessory proteins may expand treatment options. Viral protein R (Vpr) is one of the promising drug targets among the HIV accessory proteins. However, the search for inhibitors continues in anti-HIV drug discovery. In this review, we summarize the naturally occurring compounds discovered from two Myanmar medicinal plants as well as their structure-activity relationships. A total of 49 secondary metabolites were isolated from *Kaempferia pulchra* rhizomes and *Picrasama javanica* bark, and the types of compounds were identified as isopimarane diterpenoids and picrasane quassinoids, respectively. Among the isolates, 7 diterpenoids and 15 quassinoids were found to be Vpr inhibitors lacking detectable toxicity, and their potencies varied according to their respective functionalities.

## Introduction

Human immunodeficiency virus (HIV) disease has remained the fifth major worldwide killer during the past decade and the second major killer in low-income countries. According to UNAIDS, globally there were 36.7 million people living with HIV in 2015, and 1.2 million people died of AIDS that year [1]. To date, the US Food and Drug Administration (FDA) has approved 26 anti-HIV drugs, including nucleoside and non-nucleoside inhibitors of reverse transcriptase, protease inhibitors, fusion inhibitors, an entry inhibitor, and HIV integrase strand transfer inhibitors [2, 3]. However, the treatment of HIV with the FDA-approved drugs is only effective in reducing viral replication, and HIV rapidly gains resistance to all known agents [4]. To circumvent this problem, combination therapy (highly active antiretroviral therapy, HAART) has proven very effective at both reducing the virus load and suppressing the emergence of resistance in a number of patients [5]. However, the patients who received HAART have developed symptoms of Cushing’s syndrome, with hyperglycemia, hyperlipidemia, centripetal fat distribution, and peripheral muscle wasting [6]. These adverse phenomena highlight the need for new antiviral agents, preferably targeting other viral components to reduce the rate of resistance and suppress viral replication even further.

Viral protein R (Vpr) is a small basic accessory protein (14 kDa) and is well conserved in HIV-1, HIV-2, and simian immunodeficiency virus (SIV) [4, 7]. Numerous investigations over the past 2 decades have shown that Vpr is a multifunctional protein. Vpr mediates many processes that aid HIV-1 infection, evasion of the immune system, and persistence in the host, thus contributing to the morbidity and mortality of AIDS. Vpr molecular functions include nuclear import of viral preintegration complex (PIC), induction of G_2_ cell cycle arrest, modulation of T cell apoptosis, transcriptional coactivation of viral and host genes, and regulation of nuclear factor kappa B (NF-κB) activity [8–11]. The numerous functions of Vpr in the viral life cycle suggest that Vpr would be an attractive target for therapeutic intervention [12, 13]. To date, only a few small molecule Vpr inhibitors that are derived from natural sources, such as medicinal plants and fungi, are available. Among the reported Vpr inhibitors, fumagillin is an isolate from fungal metabolites [14]. Damnacanthal and quercetin are constituents of noni and a well-known plant flavonoid, respectively [15, 16]. Vipirinin is a coumarin-based synthetic compound [17]. The structures of the reported Vpr inhibitors are quite different from each other at the scaffold level, and thus the further discoveries of structurally distinct Vpr inhibitor candidates are eagerly expected from natural resources. Consequently, the anti-Vpr activities of crude extracts from Myanmar medicinal plants were evaluated to identify Vpr inhibitors. Herein, we compiled the naturally occurring Vpr inhibitors from *Kaempferia pulchra* rhizomes [18–21] and *Picrasma javanica* bark [22–24].

### Establishment of the screening system

Since Vpr function is crucial for the development of AIDS and Vpr is proposed to be a possible target molecule of anti-AIDS drugs, several groups have established assay systems to screen for Vpr inhibitors. Shimura and co-workers established a cell line [MIT (multinuclear cell induced by tetracycline)-23], in which Vpr-induced cell cycle perturbation could be manipulated by a tetracycline promoter, and identified quercetin as a Vpr inhibitor in 1999 [16]. Watanabe and co-workers established HeLa-derived cell lines (MT-Vpr 1 cells and MT-Vpr 2 cells) that allow conditional expression of Vpr and examined the mechanism of cell death following Vpr expression. They identified fumagillin as an antagonist of Vpr-mediated growth inhibition in yeast cells in 2006 [14]. In 2011, Ong and co-workers identified vipirinin, which inhibits the cell cycle arrest activity of Vpr in yeast and the Vpr-dependent viral infection of human macrophages [17]. In the same year, Kamata and co-workers reported that damnacanthal, a component of noni, is a specific inhibitor of Vpr-associated cell death with no effect on cell cycle arrest [15].

On the basis of the previously reported assay systems, we have developed a tetracycline-inducible expression system that consists of two key expression vectors, pcDNA4/TO and pcDNA6/TR. Briefly, Vpr expression plasmids were first constructed and then inserted into TREx-Hela cells to establish TREx-HeLa-Vpr cells. In this assay system, the addition of tetracycline leads to the expression of Vpr, which causes the death of TREx-HeLa cells. In contrast, Vpr-induced cell death does not occur in the presence of a Vpr inhibitor. This assay system is feasible for the preliminary screening of extracts or compounds possessing anti-Vpr activity for HIV infection as compared to the use of the virus. The detailed methods are described in our previous reports [21, 24]. The anti-Vpr activity is monitored by the cell proliferation (%), occurring because of the inhibitory effects of the tested compounds on the expression of Vpr.

### *Kaempferia pulchra* and *Picrasma javanica* as folk medicines

*Kaempferia pulchra* Ridl. is a perennial herb of the Zingiberaceae family. It is cultivated in some tropical countries, including Myanmar, Indonesia, Malaysia, and Thailand. It is commonly known as “Shan-pan-oot” in Myanmar and has been extensively used for cough, blood stimulation, carminative, quenching heat, deodorant, urinary tract infection, diuretic, and diabetes mellitus purposes [25]. It reportedly possesses antiinflammatory and antitumor activities [26, 27]. The rhizomes have been locally used for self-medication by cancer and AIDS patients. Sandaracopimaradiene diterpenoids and ethyl 4-methoxy-*trans*-cinnamate have been detected in extracts from the plant grown in Thailand [26, 28].

*Picrasma javanica* Blume is a medium-sized tree belonging to the Simaroubaceae family. The plants of the Simaroubaceae family are known to contain structurally diverse and biologically active quassinoids, with significant cytotoxic and antimalarial activities. *P. javanica* is wildly distributed in the tropical regions of Asia, including Myanmar, Indonesia, and India. It is known as “Nann-paw-kyawt” in Myanmar and has been extensively used for self-medication by malaria, cancer, and AIDS patients. Decoctions of its bark are used in folk medicine as a febrifuge and a substitute for quinine. Numerous quassinoids and alkaloids have been reported as phytoconstituents of *P. javanica*  [29–46].

### Studies on the phytochemical constituents and the Vpr inhibitors in *Kaempferia pulchra* rhizomes

**Fig. 1:**
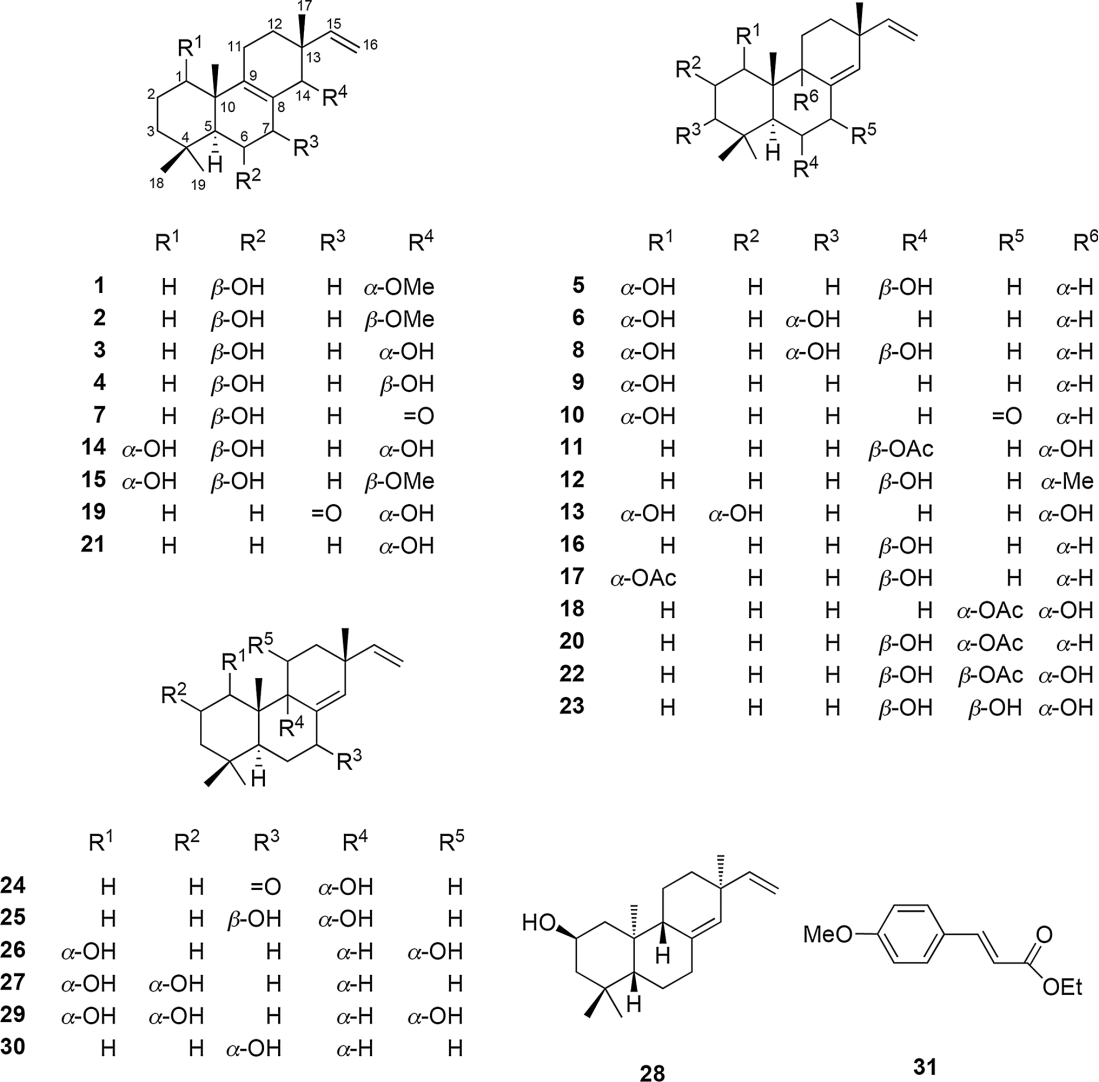
Structures of compounds isolated from *K. pulchra* rhizomes

**Fig. 2:**
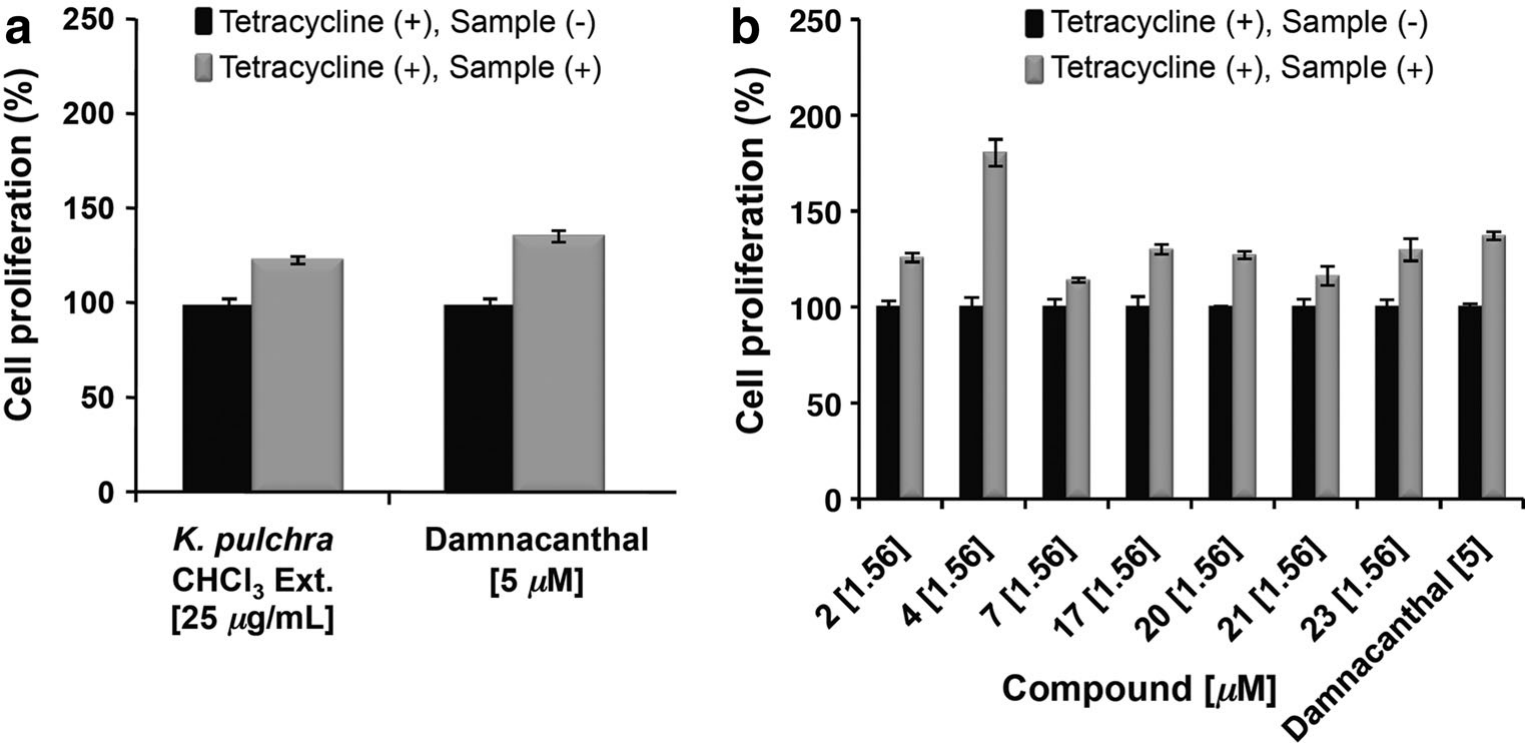
The inhibitory effects of **a** the CHCl_3_-soluble extract of *K. pulchra* and **b** the potent compounds and the positive control damnacanthal against the expression of Vpr in TREx-HeLa-Vpr cells

On the basis of the utilization of *Kaempferia pulchra* Ridl. rhizomes as a treatment for AIDS patients in Myanmar, the rhizomes were collected from Pindaya Township, Shan State, Myanmar, in September 2013. The dried rhizomes (500 g) were extracted in CHCl_3_ with sonication (1L, 90 min, ×3) at 35° C, and the solvent was evaporated under reduced pressure to give 30 g of extract. The chloroform extract (30 g) was chromatographed on silica gel with an EtOAc−*n*-hexane solvent system into seven fractions. A series of chromatographic separations of these fractions afforded 31 compounds, including 23 new isopimarane diterpenoids, kaempulchraols A−W (**1**−**23**) [18–21], and seven known analogs, such as 9*α*-hydroxyisopimara-8(14),15-dien-7-one (**24**) [47], 7*β*,9*α*-dihydroxypimara-8(14),15-diene (**25**) [48], (1*S*,5*S*,9*S*,10*S*,11*R*,13*R*)-1,11-dihydroxypimara-8(14),15-diene (**26**) [49], sandaracopimaradien-1*α*,2*α*-diol (**27**) [26], (2*R*)-*ent*-2-hydroxyisopimara-8(14),15-diene (**28**) [50], (1*R*,2*S*,5*S*,9*S*,10*S*,11*R*,13*R*)-1,2,11-trihydroxypimara-8(14),15-diene (**29**) [49], 7*α*-hydroxyisopimara-8(14),15-diene (**30**) [51], and ethyl 4-methoxy-*trans*-cinnamate (**31**) [26] (Fig. 1). The anti-Vpr activities of the crude extract of *K. pulchra* and its isolates were studied using TREx-HeLa-Vpr cells. Damnacanthal, a reported potent Vpr inhibitor, was used as a positive control. The chloroform-soluble extract of *K. pulchra* showed anti-Vpr activity at 25 *μ*g/mL (124% cell proliferation) (Fig. 2a). Among the tested compounds, only kaempuchraols B (**2**), D (**4**), G (**7**), Q (**17**), T (**20**), U (**21**), and W (**23**) inhibited the expression of Vpr at concentrations ranging from 1.56 to 6.25 *μ*M (Table 1). Among the doses of the isolated diterpenoids, the treatment with 1.56 *μ*M was the most effective. Thus, we discussed the detailed inhibitory activities of this dose [cell proliferation (%) with 1.56 *μ*M dose: 126 (**2**), 180 (**4**), 114 (**7**), 130 (**17**), 127 (**20**), 116 (**21**), 130 (**23**)] (Fig. 2b) and found that their inhibition potencies are comparable to those of 5 *μ*M of the positive control, damnacanthal (137% cell proliferation). The kaempulchraols B (**2**), D (**4**), G (**7**), and U (**21**) are classified as isopimara-8(9),15-dienes, whereas the kaempulchraols Q (**17**), T (**20**), and W (**23**) are isopimara-8(14),15-dienes.

**Table 1:** The inhibitory effects of the CHCl_3_-soluble extract and the active isolated compounds of *K. pulchra* and the positive control damnacanthal against the expression of Vpr in TREx-HeLa-Vpr cells (Data are means ± SD from three different experiments)

Samples	Cell proliferation (%) ± SD			
	Tetracycline (+), **Sample** (+)			Tetracycline (+), **Sample** (−)
	[6.25]^a^	[3.12]^a^	[1.56]^a^	
**2**	114 ± 4	119 ± 3	126 ± 2	100 ± 3
**4**	157 ± 7	170 ± 7	180 ± 7	100 ± 5
**7**	98 ± 1	106 ± 1	114 ± 1	100 ± 4
**17**	105 ± 4	116 ± 5	130 ± 2	100 ± 5
**20**	96 ± 5	114 ± 7	127 ± 2	100 ± 1
**21**	104 ± 4	106 ± 3	116 ± 5	100 ± 4
**23**	96 ± 5	111 ± 5	130 ± 6	100 ± 4
CHCl_3_-soluble extract^*b*^	124 ± 2			100 ± 2
Damnacanthal^*c*^	137 ± 2			100 ± 2

^a^The treated concentration in *μ*M

^b^The treated concentration was 25 *μ*g/mL

^c^The treated concentration was 5 *μ*M

### Studies on the phytochemical constituents and Vpr inhibitors of *Picrasma javanica* bark

**Fig. 3:**
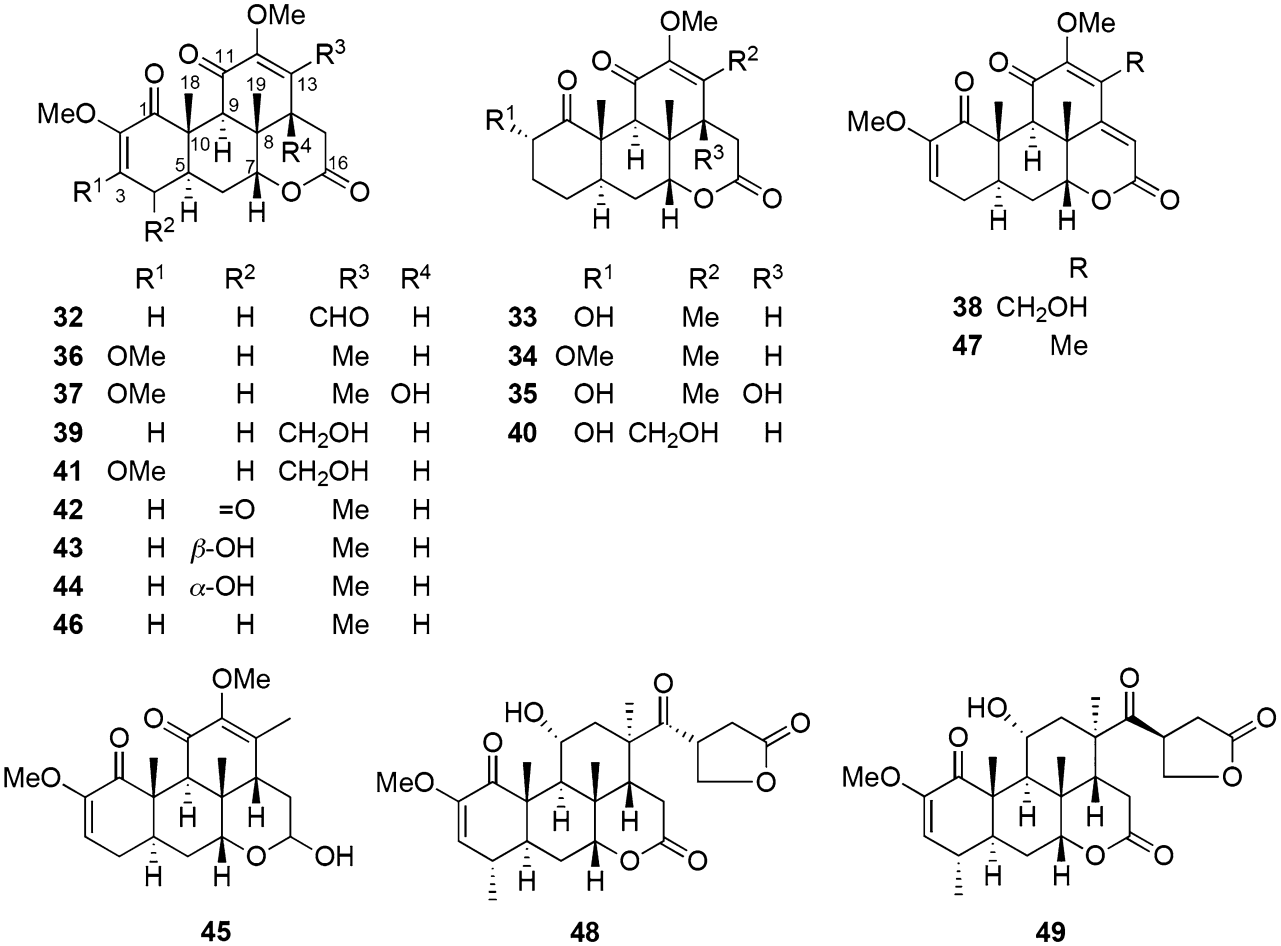
Structures of compounds isolated from *P. javanica* bark

In addition to *K. pulchra,* the dried bark of *P. javanica* was collected from Kayin State, Myanmar, in May 2014 on the basis of the utilization of the plant for AIDS patients in Myanmar. The dried bark (550 g) was extracted in CHCl_3_ with sonication (2 L, 90 min, ×3) at 30 °C, and the solvent was evaporated under reduced pressure to yield a CHCl_3_ extract (17 g). The CHCl_3_ extract (15 g) was chromatographed on silica gel with EtOAc−*n*-hexane and EtOAC−MeOH solvent systems to give eight fractions. The separation of fractions 7 and 8, using a combination of various chromatographic methods, afforded 13 new picrajavanicins A−M (**32**−**44**) [22, 23] and 5 known ones including javanicins B (**45**) [38], F (**46**) [40], and I (**47**) [37], picrasin A (**48**), and 2′-isopicrasin A (**49**) [52] from the CHCl_3_-soluble extract of *P. javanica* (Fig. 3). The structures of all isolated compounds were elucidated using extensive spectroscopic techniques, including X-ray diffraction analysis.

**Fig. 4:**
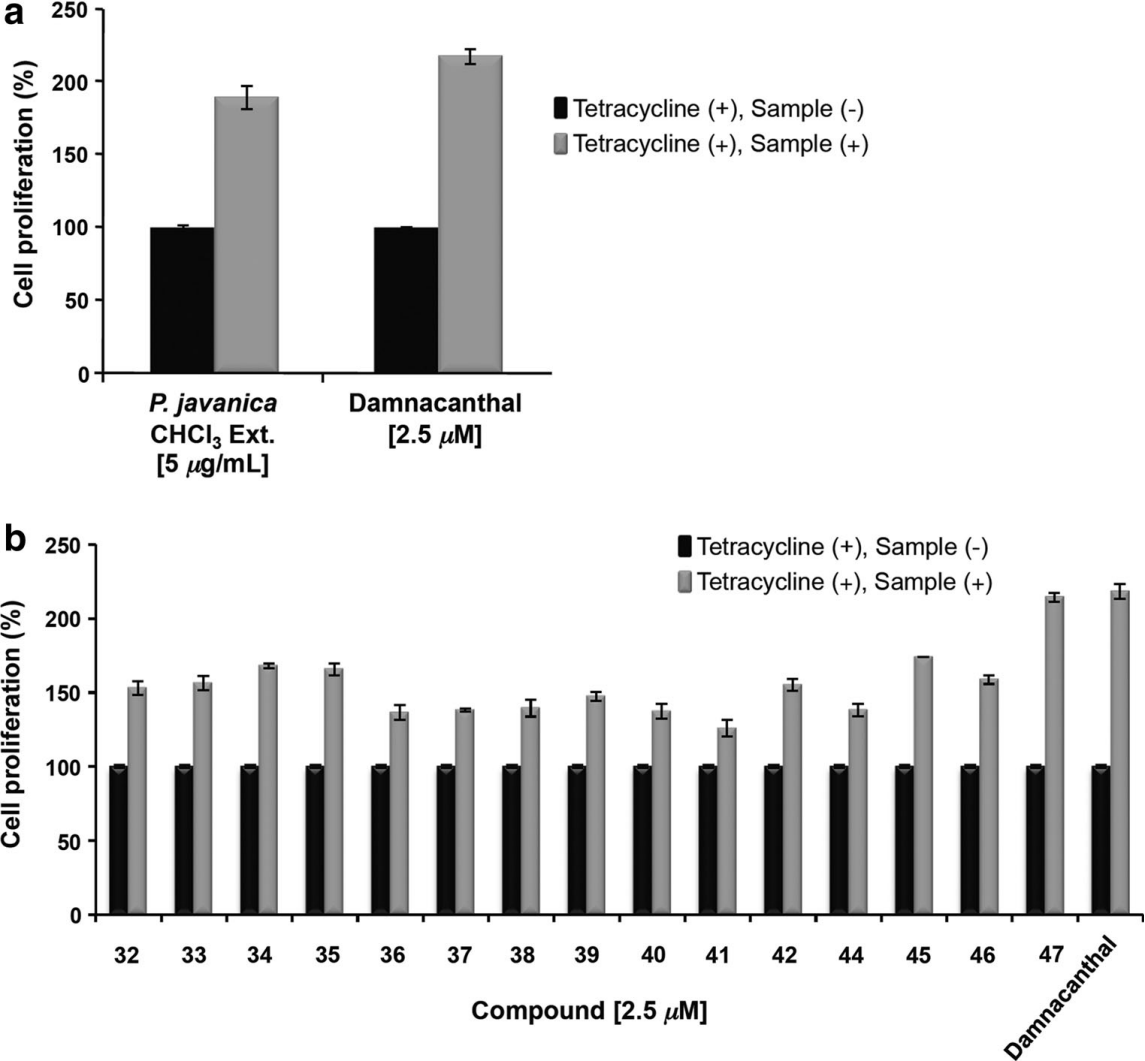
The inhibitory effects of **a** the CHCl_3_-soluble extract of *P. javanica* and **b** the isolated compounds and the positive control damnacanthal against the expression of Vpr in TREx-HeLa-Vpr cells

In the preliminary screening, the chloroform-soluble extract of *P. javanica* displayed anti-Vpr activity at 5 *μ*g/mL (189% cell proliferation) (Fig. 4a). Based on the yields of the isolates, the anti-Vpr activities of picrajavanicins A−K (**32**−**42**) and M (**44**), and javanicins B (**45**), F (**46**), and I (**47**) were evaluated against the TREx-HeLa-Vpr cells at concentrations of 1.25, 2.5, and 5 *μ*M (Table 2). Among the treatment doses, the 2.5 *μ*M dose was found to be the most effective. The cell proliferation percentages in the presence of 2.5 *μ*M of the treated compounds were **32** (153%), **33** (156%), **34** (168%), **35** (166%), **36** (136%), **37** (138%), **38** (140%), **39** (147%), **40** (137%), **41** (126%), **42** (155%), **44** (138%), **45** (174%), **46** (159%), and **47** (214%). The order of potency at 2.5 *μ*M was significant and observed as **47** > **45** > **34**, **35** > **32**, **33**, **42**, **46** > **39** > **36**, **37**, **38**, **40**, and **44** > **41** (Fig. 4b). The types of quassinoids evaluated for the anti-Vpr activity can be classified as 2,12-diene-1,11,16-trione-2,12-dimethoxy-18-norpicrasane quassinoids (**32**, **39**, **42**, **44**, **46**), 12-ene-1,11,16-trione-12-methoxy-18-norpicrasane quassinoids (**33**, **34**, **35**, **40**), 2,12-diene-1,11,16-trione-2,3,12-trimethoxy-18-norpicrasane quassinoids (**36**, **37**, **41**), 2,12,14-triene-1,11,16-trione-2,12-dimethoxy-18-norpicrasane quassinoids (**38**, **47**), and a 2,12-diene-1,11-dione-2, 12-dimethoxy-16-hydroxy-18-norpicrasane quassinoid (**45**).

**Table 2:** The inhibitory effects of the CHCl_3_-soluble extract and isolated compounds of *P. javanica* and the positive control damnacanthal against the expression of Vpr in TREx-HeLa-Vpr cells (data are means ± SD from three different experiments)

Samples	Cell proliferation (%) ± SD			
	Tetracycline (+), **Sample** (+)			Tetracycline (+), **Sample** (−)
	[1.25]^a,b^	[2.5]^a,b^	[5]^a,b^	
**32**	131 ± 0	153 ± 5	152 ± 3	100 ± 1
**33**	156 ± 6	156 ± 5	158 ± 2	100 ± 1
**34**	165 ± 7	168 ± 2	173 ± 4	100 ± 1
**35**	162 ± 4	166 ± 4	170 ± 2	100 ± 1
**36**	112 ± 6	136 ± 5	134 ± 5	100 ± 1
**37**	141 ± 2	138 ± 1	139 ± 4	100 ± 1
**38**	132 ± 4	140 ± 6	141 ± 2	100 ± 1
**39**	148 ± 7	147 ± 3	150 ± 2	100 ± 1
**40**	102 ± 7	137 ± 5	135 ± 9	100 ± 1
**41**	139 ± 3	126 ± 6	127 ± 4	100 ± 1
**42**	117 ± 9	155 ± 4	155 ± 5	100 ± 1
**44**	140 ± 4	138 ± 4	141 ± 3	100 ± 1
**45**	170 ± 4	174 ± 0	178 ± 2	100 ± 1
**46**	157 ± 4	159 ± 3	163 ± 6	100 ± 1
**47**	175 ± 1	214 ± 3	216 ± 4	100 ± 1
CHCl_3_-soluble extract	150 ± 5	181 ± 7	189 ± 8	100 ± 1
Damnacanthal^c^	203 ± 0	218 ± 5	219 ± 6	100 ± 1

^a^The treated concentration for compounds (*μ*M)

^b^The treated concentration for extract (*μ*g/mL)

^c^Positive control

### Structure-activity relationships of isopimarane diterpenoids and of picrasane quassinoids

Since the isolated 7 diterpenoids and 15 quassinoids exhibited Vpr inhibitory activities without detectable toxicities, the structure-activity relationships (SARs) for each type of isolate were studied. The SARs among diterpenoids revealed that the presence of the *β*–OH groups at C-6 and C-14 on the isopimara-8(9),15-diene skeleton increased the inhibition potency, i.e., more viable cells, because of the inhibitory effect of the treated compounds on the expression of Vpr [cell proliferation (%): 180 (**4**)] as compared to the treatments with **2** and **7** [cell proliferation (%): 126 (**2**); 114 (**7**)]. Thus, among the isopimara-8(9),15-dienes, the presence of either a methoxy or carbonyl group led to a decrease in the inhibition potency. Since **17**, **20**, and **23** exhibited similar inhibitory effects at 1.56 *μ*M, it can be concluded that the presence of an *α*–OAc or *β*–OAc group at either C-1 or C-7 on the 6*β*-hydroxy-isopimara-8(14),15-diene skeleton is important for the inhibition of Vpr expression.

Among the tested picrasane quassinoids, javanicin I (**47**) was the most potent inhibitor, with potency comparable to that of the positive control, damnacanthal [cell proliferation (%): 214 (**47**); 218 (damnacanthal)]. SAR studies between picrajavanicins G (**38**) and I (**47**) suggested that the presence of the methyl group at C-13 is crucial for exhibiting significant anti-Vpr activity in the 2,12,14-triene-1,11,16-trione-2,12-dimethoxy-18-norpicrasane quassinoids [cell proliferation (%): 140 (**38**)]. It should be noted that javanicin I (**47**) is the least polar compound among the isolates from the CHCl_3_ extract. This finding suggested that the polarity of the C-13 side chain is related to the Vpr inhibitory effect.

SAR studies of the 12-ene-1,11,16-trione-12-methoxy-18-norpicrasane quassinoids (**33**, **34**, **35**, **40**) also revealed that the methyl group at C-13 is an important functionality, whereas the hydroxymethyl group at C-13 is not as effective for the inhibition of the Vpr expression [cell proliferation (%): 156 (**33**) > 137 (**40**)]. Furthermore, the presence of either a hydroxy or methoxy group at C-2 did not influence the activity [cell proliferation (%): 168 (**34**); 166 (**35**)], while the presence of the hydroxy group at C-14 was effective for the activity [cell proliferation (%): 166 (**35**) > 156 (**33**)].

Similarly, among the 2,12-diene-1,11,16-trione-2,12-dimethoxy-18-norpicrasane quassinoids (**32**, **36, 37**, **39**, **41**, **42**, **44**, **46**), the C-13 methylated **46** possessed the most potent activity among its analogs. Interestingly, the substitutions of the methyl group with the aldehyde and hydroxymethyl groups at C-13 weakened the activity [cell proliferation (%): 159 (**46**) > 153 (**32**) > 147 (**39**)]. In addition, the methoxy group at C-3 in the structure led to a decrease in the anti-Vpr activity [cell proliferation (%): 159 (**46**) > 136 (**36**); 138 (**37**) > 126 (**41**)]. Furthermore, comparisons of the structures of **46** and the C-4 substituted 2,12-diene-1,11,16-trione-2,12-dimethoxy picrasane quassinoids (**42** and **44**) revealed that the carbonyl group at C-4 favored the activity compared to the hydroxy group [cell proliferation (%): 159 (**46**) > 155 (**42**) > 138 (**44**)]. However, the substitutions at C-4 with the functional groups are rather ineffective for the potent activity of the quassinoids.

The SARs of the 2,12-diene-1,11,16-trione-2,3,12-trimethoxy-18-norpicrasane quassinoids (**36**, **37**, **41**) revealed that the presence of the hydroxy group at C-14 increased the activity, while the substitution of the hydroxymethyl group weakened the activity [cell proliferation (%): 138 (**37**) > 136 (**36**) > 126 (**41**)]. However, the anti-Vpr activity of **37** was weaker than that of **46** [cell proliferation (%): 159 (**46**) > 138 (**37**)]. Thus, in the case of the 2,12-diene-1,11,16-trione-2,12-dimethoxy-18-norpicrasane quassinoids, the presence of the methyl group at C-13, the hydroxy or carbonyl groups at C-4, and the hydroxy group at C-14, and the absence of the methoxy group at C-3, are important for the potent activity.

Furthermore, the C-13 methylated 2,12-diene-1,11-dione-2,12-dimethoxy-18-norpicrasane quassinoid, **45**, is the second most potent Vpr inhibitor among the isolates [cell proliferation (%): 214 (**47**) > 174 (**45**)]. The differences between the structures of **45** and the most potent inhibitor, **47**, are the substitutions of the double bond with a single bond between C-14 and C-15 and of the carbonyl group with the hydroxy group at C-16. Hence, the presence of the hydroxy group at C-16 may serve as an effective functional group that increases the anti-Vpr activity. A comprehensive assessment of the results obtained between the tested quassinoids suggests that the presence of the methyl group at C-13 is an important factor and the substitution of the carbonyl group with a hydroxyl group at C-16 of **47** increases the anti-Vpr activity.

### Antiproliferative activities of isolated isopimarane diterpenoids and picrasane quassinoids

Some of the isolates did not show any Vpr inhibitory activity. However, the rhizomes of *K. pulchra* and the bark of *P. javanica* have been locally used for self-medication by cancer patients [25]. Therefore, the antiproliferative activities of the isolated compounds (**1**–**49**) were evaluated against a panel of five human cancer cell lines, including A549 (human lung cancer), HeLa (human cervical cancer), PANC-1 and PSN-1 (human pancreatic cancer), and MDA-MB-231 (human breast cancer) using the CCK-8 assay. Among the tested compounds, **39**, **48**, and **49** were selectively active against HeLa and PANC-1 cells, whereas **41**, **44** selectively inhibited the proliferation of PANC-1 cells (Tables 3, 4). It is interesting that all of the potent anti-Vpr diterpenoids and quassinoids lacked cytotoxic effects against the tested human cancer cell lines. These results provided convincing evidence that the Vpr inhibitors from the present study might be capable of selectively targeting the expression of Vpr with no toxicity.

**Table 3:** Antiproliferative activities (IC_50_ *μ*M) of compounds **1**−**31** isolated from *K. pulchra* against a panel of five human cancer cell lines

Compounds	Cell line^a^				
	A549	HeLa	PANC-1	PSN-1	MDA-MB-231
**1**	>100	88.7	54.4	56.5	>100
**2**	32.9	>100	25.4	23.2	71.4
**3**	59.1	60.3	>100	44.0	>100
**4**	36.7	>100	32.0	44.1	70.0
**5**	49.8	>100	>100	44.3	48.2
**6**	75.9	79.9	76.0	12.3	63.4
**7**	46.1	64.2	>100	47.9	62.8
**8**	>100	>100	>100	>100	>100
**9**	55.8	58.2	70.2	39.9	79.6
**10**	71.6	>100	>100	70.3	>100
**11**	33.1	>100	>100	>100	>100
**12**	72.3	>100	39.9	22.6	73.6
**13**	45.8	28.4	>100	45.5	>100
**14**	>100	>100	>100	>100	>100
**15**	>100	>100	>100	>100	>100
**16**	69.5	74.7	93.5	30.0	>100
**17**	70.9	36.7	59.8	38.4	70.9
**18**	78.6	61.1	73.7	63.5	78.6
**19**	>100	73.4	84.8	90.1	>100
**20**	44.8	31.9	45.8	29.6	44.8
**21**	>100	>100	>100	>100	>100
**22**	>100	>100	>100	>100	>100
**23**	>100	>100	>100	>100	>100
**24**	>100	>100	>100	>100	>100
**25**	>100	>100	>100	>100	>100
**26**	44.4	67.5	>100	42.2	60.8
**27**	43.0	64.9	>100	30.4	68.3
**28**	33.1	88.4	52.8	21.4	50.0
**29**	93.1	59.4	91.7	99.3	>100
**30**	43.4	40.0	74.7	31.2	87.8
**31**	>100	>100	>100	>100	>100
5-Fluorouracil^b^	2.8	5.8	3.7	4.4	5.2

^a^ *A549* human lung cancer, *HeLa* human cervical cancer, *PANC-1, PSN-1* human pancreatic cancer, *MDA-MB-231* human breast cancer

^b^Positive control

**Table 4:** Antiproliferative activities (IC_50_ *μ*M) of compounds **32**−**49** isolated from *P. javanica* against a panel of five human cancer cell lines

Compounds	Cell line^a^				
	A549	HeLa	PANC-1	PSN-1	MDA-MB-231
**32**	97.1	>100	>100	>100	>100
**33**	>100	>100	>100	>100	>100
**34**	>100	>100	>100	>100	>100
**35**	>100	>100	>100	>100	>100
**36**	>100	>100	>100	>100	>100
**37**	>100	>100	>100	>100	49.2
**38**	>100	>100	>100	>100	>100
**39**	> 100	9.50	4.33	>100	>100
**40**	> 100	>100	17.4	>100	>100
**41**	>100	>100	9.95	>100	>100
**42**	>100	>100	3.25	>100	>100
**43**	>100	>100	8.48	>100	>100
**44**	>100	37.7	7.37	88.1	>100
**45**	>100	>100	>100	>100	22.2
**46**	>100	>100	>100	>100	22.2
**47**	>100	>100	>100	>100	54.1
**48**	>100	10.2	5.60	>100	>100
**49**	>100	3.98	3.90	>100	>100
5-Fluorouracil^b^	9.0	6.4	0.4	8.7	1.1

^a^ *A549* human lung cancer, *HeLa* human cervical cancer, *PANC-1, PSN-1* human pancreatic cancer, *MDA-MB-231* human breast cancer

^b^Positive control

### Future perspectives

Isopimarane diterpenoids and picrasane quassinoids are naturally occurring Vpr inhibitor candidates that are chemically quite distinct from the previously reported ones [14–17]. Further structurally interesting Vpr-inhibitors might be expected from structural modifications of the isopimarane diterpenoids and picrasane quassinoids. However, while numerous Vpr inhibitors have been identified over the past few years, structurally distinct compounds from natural sources are still necessary for understanding the structure-activity relationships completely. The action mechanism of each potent Vpr inhibitor against TREx-HeLa-Vpr should be clarified. Understanding the detailed mechanisms with Vpr and different types of natural products may facilitate the development of new drugs for the treatment of AIDS.
